# IUC25693-90 Real-world effectiveness of pembrolizumab plus axitinib in metastatic renal cell carcinoma in Panama

**DOI:** 10.1093/oncolo/oyag286.022

**Published:** 2026-08-21

**Authors:** J Pinto, A Cardenas

**Affiliations:** Head of Oncology Unit, Hospital Anita Moreno. Los Santos, Panama - Panama; MD, Oncology Unit, Hospital Anita Moreno, Los Santos. Panama - Panama

## Abstract

**Background:**

To evaluate the effectiveness and safety of pembrolizumab plus axitinib as first-line therapy for metastatic renal cell carcinoma (mRCC) in a national real-world cohort. This study is the first from Panama and the first from Central America to address this treatment strategy.

**Methods:**

We conducted a retrospective, multicenter observational cohort study using electronic medical records from the National Oncology Network of Panama (2020–2024). Eligible patients were ≥18 years old, had histologically confirmed mRCC, had documented metastatic disease, and received pembrolizumab plus axitinib as first-line therapy with at least 3 months of follow-up. Patients with prior immunotherapy, incomplete records, or concurrent experimental treatments were excluded. Overall survival (OS) and progression-free survival (PFS) were estimated using the Kaplan–Meier method. Multivariable Cox regression was used to identify prognostic factors, including age, ECOG performance status, IMDC risk group, liver metastases, and treatment modifications. Statistical analyses were conducted in Stata® version 19.

**Results:**

A total of 103 patients were included (median age 65 years; 73.8% male). Most patients had an ECOG performance status of 0–1 and intermediate or poor IMDC risk. The median follow-up was 18.9 months. The median PFS was 24.2 months (95% CI, 17.2–not reached), and the median OS was 31.7 months (95% CI, 23.4–not reached). The objective response rate was 36.9%, the disease control rate was 80.6%, and the median duration of response was 13.8 months. In multivariable analysis, objective response was independently associated with improved outcomes (PFS: HR 0.45, 95% CI 0.28–0.72; OS: HR 0.38, 95% CI 0.22–0.65), whereas IMDC poor risk was associated with worse survival (OS: HR 2.10, 95% CI 1.25–3.52). The most frequent adverse events were hypothyroidism, transaminitis, and diarrhea. Grade ≥3 adverse events occurred in 20.4% of patients, most commonly hypertension, transaminitis, and diarrhea.

**Conclusions:**

This first Central American real-world study demonstrates that pembrolizumab plus axitinib yields clinically meaningful outcomes and a manageable safety profile in mRCC. Survival outcomes align with previously reported real-world series, supporting the external validity of this combination in resource-constrained settings. IMDC risk remains a key prognostic factor for clinical decision-making.

